# First report of OXA-48 carbapenemase-producing Escherichia coli in Taiwan

**DOI:** 10.1186/2047-2994-4-S1-P126

**Published:** 2015-06-16

**Authors:** Y Jao, P-S Lee, C-T Hung, C-F Wang, MH Hsieh, C-Y Lin, Y-H Chen, P-L Lu

**Affiliations:** 1 Department of Infection Control, Kaohsiung Medical University Hospital, Kaohsiung Medical University, Kaohsiung, Taiwan, Province of China; 2 Clinical Microbiology Laboratory, Kaohsiung Medical University Hospital, Kaohsiung Medical University, Kaohsiung, Taiwan, Province of China; 3 Division of Infectious Diseases, Department of Internal Medicine, Kaohsiung Medical University Hospital,Kaohsiung Medical University, Kaohsiung, Taiwan, Province of China

## Introduction

Carbapenems are the last-line antibiotic in treating Enterobacteriaceae because its broad-spectrum antibacterial ability, high bactericidal activity and good stability to β-lactamases. However, carbapenem-resistant Enterobacteriaceae (CRE) infection increased all over the world in recent years. The challenge of carbapenems in treating Enterobacteriaceae is increasing.

## Objectives

A 55-year old woman who had been diagnosed with cancer and had received multiple courses of chemotherapy in Vietnam, visited our hospital for a second opinion in June 2014.

Left breast tumor with non-discharge necrotic tissue was presented at admission. The laboratory examination demonstrated WBC 6300/uL, Hb 12.1g/dL and PLT 103000/uL. Specimen from left breast wound grew *Escherichia coli*, which resistant to carbapenems, broad-spectrum β-lactams and fluoroquinolones. There was no inflammation reaction caused by the pathogen. During hospitalization, she did not receive antimicrobial therapy effective to the pathogen.

## Methods

We found that this *Escherichia coli* had the presentation of carbapenemase via modified Hodge test. Polymerase chain reaction and DNA sequencing was performed to test if Carbapenemase (blaKPC, blaNDM, blaIPM, blaVIM, blaOXA) or β-lactamases (SH, TEM, OXA, GES, CTX-M) presents.

## Results

Resistance genes of blaOXA-48 and CTX-M-1-group were found. Besides, this *Escherichia coli* also presented with loss of outer membrane protein A and F. With multilocus sequence typing (MLST) analysis, this *Escherichia coli* is ST-405.

## Conclusion

To our knowledge, this is the first OXA-48 carbapenemase producing *Escherichia coli* isolated in Taiwan, though it’s originated from Vietnam. It’s resistance profile is similar to other OXA-48 carbapenemase producing *Escherichia coli* isolated from Japan and France. According to this finding, *Escherichia coli* acquiring OXA-48 carbapenemase may had spread to Southeastern Asia. In order to prevent the transmission of CRE, the detection of carbapenemase in Enterobacteriaceae is important.

## Disclosure of interest

None declared.

